# Novel Patched 1 mutations in patients with nevoid basal cell carcinoma syndrome – case report

**DOI:** 10.3325/cmj.2015.56.63

**Published:** 2015-02

**Authors:** Vesna Škodrić-Trifunović, Mihailo Stjepanović, Živorad Savić, Miroslav Ilić, Ivana Kavečan, Jadranka Jovanović Privrodski, Vesna Spasovski, Maja Stojiljković, Sonja Pavlović

**Affiliations:** 1 School of Medicine, University of Belgrade, Belgrade, Serbia; 2 Clinic of Pulmonology, Clinical Center of Serbia, Belgrade, Serbia; 3 Center of Radiology and Magnetic Resonance, Clinical Center of Serbia, Belgrade, Serbia; 4 Department of Maxillofacial and Oral Surgery, Clinical Center of Vojvodina, Medical Faculty, University of Novi Sad, Novi Sad, Serbia; 5 Center for Medical Genetics, Institute for Children and Youth Health Care of Vojvodina, Medical Faculty, University of Novi Sad, Novi Sad, Serbia; 6 Institute of Molecular Genetics and Genetic Engineering, University of Belgrade, Belgrade, Serbia

## Abstract

Nevoid basal cell carcinoma syndrome (Gorlin syndrome) is a rare autosomal dominant disorder characterized by numerous basal cell carcinomas, keratocystic odontogenic tumors of the jaws, and diverse developmental defects. This disorder is associated with mutations in tumor suppressor gene Patched 1 (*PTCH1*). We present two patients with Gorlin syndrome, one sporadic and one familial. Clinical examination, radiological, and CT imaging, and mutation screening of *PTCH1* gene were performed. Family members, as well as eleven healthy controls were included in the study. Both patients fulfilled the specific criteria for diagnosis of Gorlin syndrome. Molecular analysis of the first patient showed a novel frameshift mutation in exon 6 of *PTCH1*gene (*c.903delT*). Additionally, a somatic frameshift mutation in exon 21 (*c.3524delT*) along with germline mutation in exon 6 was detected in tumor-derived tissue sample of this patient. Analysis of the second patient, as well as two affected family members, revealed a novel nonsense germline mutation in exon 8 (*c.1148 C>A*).

Nevoid basal cell carcinoma syndrome (NBCCS; Gorlin syndrome; MIM 109400) is a rare autosomal-dominant disorder with the prevalence ranging from 1/57 000 to 1/256 000 and a male-to-female ratio of 1:1 (1,2). It is characterized by a multiple basal cell carcinomas (BCCs), developmental and skeletal anomalies, keratocystic odontogenic tumors (KCOT) of the jaws, and a predisposition to a variety of other tumors (3). NBCCS may affect multiple organ systems, such as skin, skeletal system, genitourinary system, and central nervous system. It is caused by mutations in the Patched 1 (*PTCH1*) gene, a tumor suppressor gene involved in Sonic hedgehog (SHH) signaling pathway (4,5). Mutations of *PTCH1* gene have also been associated with sporadic carcinomas, such as keratocystic odontogenic tumor (KCOT) (6), medulloblastoma (7), esophageal squamous cell carcinoma (8), and some benign tumors, such as ovarian and cardiac fibromas. To date, over 230 *PTCH1* germline mutations associated with NBCCS have been reported (9), which are transmitted in an autosomal-dominant way with high penetrance and variable expressivity (10-12). We present two cases with Gorlin syndrome, one sporadic and one familial. Molecular diagnosis in patients and family members was established by molecular genetic analysis.

## Patients and samples

Our study was conducted according to the guidelines of the Helsinki Declaration of 1964, as revised in 2000. One sporadic and one familial case (involving mother and two sons) were diagnosed as Gorlin syndrome according to Kimonis et al (13) at the Clinic of Pulmonology, Clinical Center of Serbia, Belgrade (case 1), and the Department of Maxillofacial and Oral Surgery, Clinical Center of Vojvodina, Novi Sad (case 2). Buccal swab and peripheral blood samples, as well as tumor tissue from BCC for the sporadic case, were collected. The study also enrolled five family members (mother, father, two sons, and brother) for sporadic case, as well as eleven ethnically matched healthy controls. Written informed consents were obtained from all patients and controls. Ethical approval was obtained from both institutions.

### DNA isolation, amplification, and sequencing analysis

Genomic DNA was extracted from buccal swab and cancer tissue (BCC) (case 1) and from buccal swab of family members. Peripheral blood was used for DNA extraction for the case 2, his family members, and controls. DNA was extracted using QIAamp DNA Blood Mini Kit (QIAGEN, Hilden, Germany) (Supplementary material(web extra material 1)). Five controls were sequenced using the Sanger method and six using TruSight One Sequencing Panel (Illumina Inc., San Diego, CA, USA).

### Case reports

**Figure 1:**
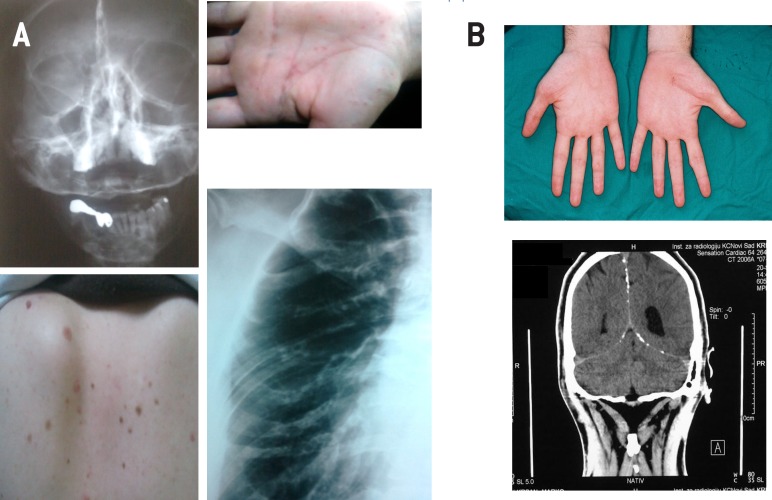
Clinical findings of the patients with Gorlin syndrome. (**A**) Patient 1: keratocystic odontogenic tumor of jaws and calcification of the falx cerebri; basal cell carcinomas on the back; palmar changes; bifid ribs. (**B**) Patient 2: palmar changes and calcification of the falx cerebri.

*Case 1*. A female patient age 49 (Figure 1A) had a long history of disease before the diagnosis was established. As a child, she underwent surgery for multiple keratocystic odontogenic tumors in the right body of the mandible. She had BCC on face, chest, and back, as well as multiple palmar and plantar pits and anomalies of the ribs (bifid ribs). At the age of 35 she started to suffer from frontal headaches (pain intensity 8/10), which lasted for several hours and then disappeared spontaneously or with analgesics. When headaches became more intense, followed by nausea, the brain CT scan was performed and showed bilateral bilamellar calcification of the falx cerebri, which led to the establishment of the diagnosis of Gorlin syndrome. Among skeletal abnormalities, pectus excavatum and kyphoscoliosis were observed, as well as facial dysmorphism (ocular hypertelorism, flat and wide nasal root). Patient had no family history of disease or affected first-degree relatives. Thus, the patient fulfilled five major and four minor criteria for diagnosis of NBCCS (Supplementary Table 1(web extra material 2)).

**Figure 2:**
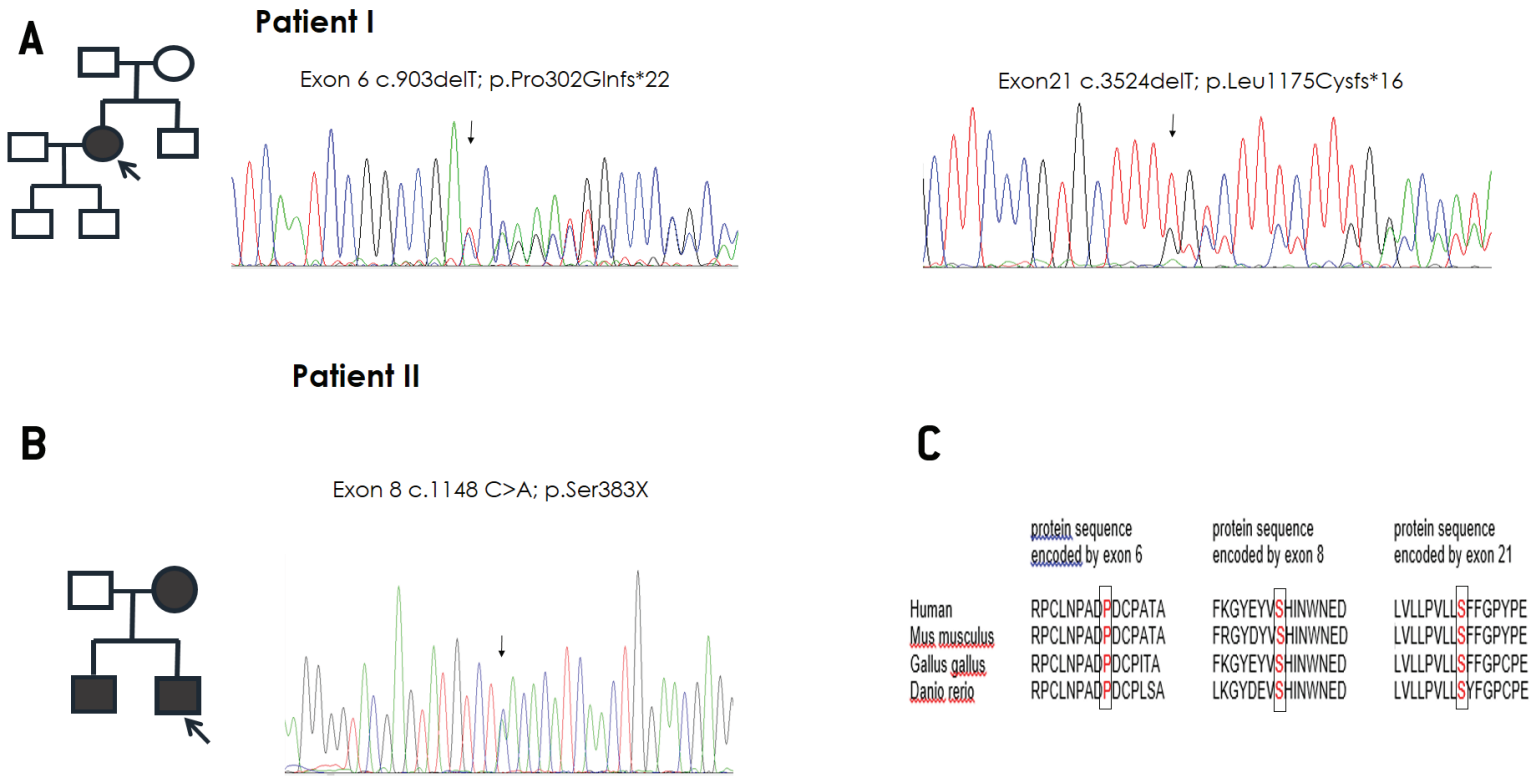
Genetic findings in patients with Gorlin syndrome. **(A)** A genetic pedigree of Patient 1. Chromatograms show germline (exon 6) and somatic (exon 21) mutations of *PTCH1* gene. **(B)** A genetic pedigree of Patient 2. Chromatogram shows germline mutation in exon 8 of *PTCH1* gene. **(**C**)** Alignment of amino acid sequences of PTCH protein. The regions of PTCH protein encoded by exon 6, exon 8, and exon 21 of different organisms are shown: *Mus musculus*, *Gallus gallus*, *Danio rerio* [GenBank: AAC50550, AAC98798, AAC59898, CAB39726, respectively]. Boxed amino acids were targeted by corresponding mutations.

*Molecular detection of PTCH1 mutations.* Direct sequencing of all coding exons of *PTCH1* gene for this patient revealed a T (thymine) base deletion at residue 903 (*c.903delT*) in exon 6 (Figure 2A). This frameshift mutation affects amino acid residue 302 (p.Pro302Glnfs*22) of PTCH protein, introduces a premature stop codon, and probably leads to synthesis of truncated protein or nonsense-mediated mRNA decay. The mutation was verified by sequencing of three independent polymerase chain reactions (PCR), in both directions. This germline mutation was present in heterozygous state, and was not detected in DNA of the patient’s relatives (mother, father, brother, and two sons) or in healthy controls.

Sequencing of the patient’s basal cell carcinoma tissue revealed a novel somatic mutation in exon 21 along with germline mutation in exon 6. The single base deletion in exon 21 (*c.3524delT*) affects amino acid residue 1175 (p.Leu1175Cysfs*16) of PTCH protein, possibly truncating the protein for 288 C-terminal amino acids (Figure 2A). The mutations were verified by sequencing of three independent PCR reactions, in both directions. None of these mutations were present in control samples.

*Case 2.* A 24-year-old man (Figure 1B) underwent a surgery for a maxillar cyst at the age of 7, and at the age of 17 a surgery for mandibular cysts. The histological examination of cysts in both cases confirmed keratocystic odontogenic tumors. The palmar round thickened nodes were noticed at the age of 15, which gradually increased over time. Macrocephaly, ocular hypertelorism, and diverse congenital and various skeletal abnormalities (high-mounted palate, prognathism, clynodactily of the fifth fingers, partial syndactyly of the second and third toes, thoracic scoliosis, numerous nevi on the skin) were also observed. Endocranial CT revealed calcification on the falx cerebri. His mother and brother had maxillar and mandibullar cysts and similar changes on the palms and numerous nevi on the skin, and were also diagnosed with Gorlin syndrome. Thus, the patient fulfilled four major and five minor criteria for the diagnosis of Gorlin syndrome (Supplementary Table 1(web extra material 2)).

*Molecular analysis of PTCH1 mutations.* Sequencing analysis of this patient revealed the presence of nonsense mutation in exon 8 at position 1148 (*c.1148 C>*A) (Figure 2B), causing a serine to stop codon substitution at amino acid residue 383 (p.Ser383*). Sequencing of other affected family members showed the presence of the same germline mutation. The mutation was verified by sequencing three independent PCR reactions, in both directions, and using additional forward primer (Supplementary Table 2(web extra material 3)). This germline mutation was present in heterozygous state in all affected family members. This mutation was not present in controls.

## Discussion

In this study we presented two cases with Gorlin syndrome. Molecular analysis of the patients showed two frameshift and one nonsense mutations in *PTCH1* gene (two germline and one somatic), all of them novel. These mutations have not previously been described in the literature. Mutations in exon 6 and exon 8 detected in our patients target the first extracellular loop of the PTCH protein, and resulting truncation of the protein would abrogate the binding of the SHH ligand. The preservation of this protein region in different species indicates its significance for protein function (Figure 2C). The mutation in exon 21 affects C- terminal portion of PTCH protein, previously shown to be involved in the apoptotic cell death during the spinal cord development (14). This part of protein is also highly conserved in various species (Figure 2C).

The pathogenesis of Gorlin syndrome has not been elucidated yet. The genotype-phenotype correlation has not been established (10,15), and no correlation between the type and the position of the mutation within the *PTCH1* gene with certain disease characteristics, such as the number and the age of onset of BCC, has been shown (16). Moreover, even patients with identical mutations differed in the extent of the clinical findings (17,18). Interestingly, mutation in exon 8, which was found in our familial case, targets the same codon as described by Scott et al (19), with the same effect on the protein level (nonsense mutation). However, our patient did not show visual impairment, as described in their publication. Findings of the recurrent mutations are rarely reported for NBCCS (17,18), making our findings intriguing.

According to the “two hit” hypothesis in the case of tumor suppressor genes, inactivation of both alleles of tumor suppressor gene is needed for tumor development (20). In the case of *PTCH1* gene, the germline mutation could represent the first hit and the deletion or the mutation of the wild type allele would represent the second hit (21,22). Our finding of both germline and somatic mutations in the tumor tissue of patient 1 could be such a case, although the possibility of somatic mosaicism cannot be ruled out.

Early diagnosis of Gorlin syndrome is of great importance for treatment of this autosomal-dominant disorder. It is also important that these patients are monitored and diagnosed by a multidisciplinary team (odontologist, dermatologist, radiologist, neurologist, etc). The high detection rate of *PTCH1* mutations in NBCCS patients enables molecular diagnostics to become a valuable tool for establishing an early diagnosis, especially in the case of atypical phenotype and for yet unaffected family members. Moreover, family members of the patients with the Gorlin syndrome should be examined to decrease the risk for developing skin tumors, like basal cell carcinoma, which could be prevented by decreased sun exposure and avoidance of x-ray imaging.

## Additional Material

Supplementary material

Supplementary teble 1
