# Supplementary material for: Novel Patched 1 mutations in patients with nevoid basal cell carcinoma syndrome – case report
Source: Croat Med J. 2015 Feb;56(1):63–7. doi: 10.3325/cmj.2015.56.63 (PMC4364350; doi:10.3325/cmj.2015.56.63)
Supplement: Supplementary material [file CroatMedJ_56_s004.pdf]

Supplementary material

*PTCH1*gene is located on chromosome 9q22.3 and consists of 24 exons, 23 of which are protein-coding exons. The *PTCH1* coding region was amplified by PCR using oligonucleotide primer pairs flanking each exon (Table 2). PCR was carried out in total reaction volume of 25µl, containing 200µM dNTPs, 0.4µM of each primer, 50ng genomic DNA and 1U KapaTaq polymerase (Kapa Biosystems, Woburn, MA, USA). Amplification was carried out for 35 cycles of 94°C for 45s, 55-57°C for 45s and 72°C for 30-45s. Molecular analysis of 23 coding exons of *PTCH1*gene was performed by direct sequencing using BigDye Terminator v3.1 Cycle Sequencing Kit (Applied Biosystems, Foster City, CA, USA) and run on 3130 Genetic Analyzer (Applied Biosystems, Foster City, CA, USA). Exon 6, 8 and 21 were sequenced in both directions, and additional F primer (TGAATGTGAACTGCGGTTGGA) was used for confirmation of mutation in exon 21.

Nucleotide and protein names were given for each mutation according to current nomenclature guidelines (http://www.hgvs.org/mutnomen/). Nucleotide numbering was based on GenBank entry [NG: 007664.1], where the A of the ATG initiation codon represents nucleotide +1. The amino acid numbering was based on GenBank entry [Q13635.2].
