# Supplementary material for: Novel Patched 1 mutations in patients with nevoid basal cell carcinoma syndrome – case report
Source: Croat Med J. 2015 Feb;56(1):63–7. doi: 10.3325/cmj.2015.56.63 (PMC4364350; doi:10.3325/cmj.2015.56.63)
Supplement: Supplementary teble 1 [file CroatMedJ_56_s005.pdf]

Table 1. Characteristics of our patients with Gorlin syndrome

Diagnostic criteria according to Kimonis et al (13)	Patient 1	Patient 2
Major symptoms		
Basal cell carcinoma (more than two or under the age of 20 years)	+	-
Keratocystic odontogenic tumor of jaws	+	+
Palmar and plantar pits (more than three)	+	+
Ectopic calcification of the falx cerebri	+	+
Abnormalities of the ribs (bifid ribs)	+	-
First degree relative with nevoid basal cell carcinoma syndrome	-	+
Minor symptoms		
Facial dysmorphism (ocular hypertelorism)	+	+
Macrocephaly (after correction for height)	-	+
Congenital malformation	+	+
Other skeletal abnormalities	+	+
Abnormality in radiological examination	+	+
Ovarian fibroma	-	-
Medulloblastoma	-	-
