# Supplementary material for: Novel Patched 1 mutations in patients with nevoid basal cell carcinoma syndrome – case report
Source: Croat Med J. 2015 Feb;56(1):63–7. doi: 10.3325/cmj.2015.56.63 (PMC4364350; doi:10.3325/cmj.2015.56.63)
Supplement: Supplementary table 2 [file CroatMedJ_56_s006.pdf]

Table 2. Primers used for amplification of *PTCH1* exons*

Exon	PCR product size	Primer sequences
Exon 1	672	F GAGCACAAGGTGGAGAAGCC<br>R CGCGCTGGCTGCACTC
Alternate Exon 1	463	F TGCCGGGTTCATTGTGTTTAC<br>R TGATGGACAGAGCAGGACTG
Exon 2	281	F CACTCCTCCCTTCTGCTTCG<br>R CTCTATCAACCGCGAGGAGG
Exon 3	286	F GAGTTTGCAGTGATTTTGCTATTC<br>R CCGCCTTACCTGCTGCTC
Exon 4+5	446	F GTGAGAAATTTTTGTCTCTGCTTTT<br>R CCTCTCCCCCGACTATTCAC
Exon 6	284	F GGCTCTTTTCATGGTCTCGTC<br>R TGTTTTGCTCTCCACCCTTC
Exon 7	226	F GCACTGGATTTTAACAAGGCATG<br>R CAGGGCATAGATTGTCCTCG
Exon 8	232	F TGGGAATACTGATGATGTGCC<br>R CATAACCAGCGAGTCTGCAC
Exon 9	213	F CATTTGGGCATTTCGCATTC<br>R CAACCAAACCAAACTCCAGC
Exon 10	208	F TGCCCCCATTGTTCTGCTTG<br>R GGACAGCAGATAAATGGCTCC
Exon 11	188	F GCATCTCGCATGTCTAATGCCAC<br>R AAGCTGTGATGTCCCCAAAG
Exon 12	211	F GACCATGTCCAGTGCAGCTC<br>R CGTTCAGGATCACCACAGCC
Exon 13	222	F AGTCCTCTGATTGGGCGGAG<br>R CCATTCTGCACCCAATCAAAAG
Exon 14	541	F AAAAATGGCAGAATGAAAGCACC<br>R CTGATGAACTCCAAAGGTTCTG
Exon 15	405	F GGAAGAGTCAGTGGTGCTCC<br>R CGCAAAAGACCGAAAGGAC
Exon 16	318	F AGGGTCCTTCTGGCTGCGAG<br>R AGCTCCCAGTGCCTTAGGTC
Exon 17	303	F GCTCTCAAGGCAGAAGTGTG<br>R GGAAGGCACCTCTGTAAGTTC
Exon 18	344	F GCTCCTAACCTGTGCCCTTC<br>R TTGACTTCCACAAAGCCCCTT
Exon 19	227	F CGCCCACTGACCACTGTGTG<br>R GAGCCAGAGGAAATGGGTTG
Exon 20	217	F AGCATTTACCAGGTGAAGTCC<br>R TTGCACACGCCTGCTTAC
Exon 21	309<br>187	F_1 TGTTCCCGTTTCCTCTTG<br>F_2 TGAATGTGAACTGCGGTTGGA<br>R GCACAGGAAACACAGCATTC
Exon 22	352	F GCAGGTAAATGGACAAGAACAC<br>R ACTACCACGGTGGGAAGACC
Exon 23	731	F CCCTTCTAACCCACCCTCAC<br>R GACACATCAGCCTTGCTC

*Nucleotide sequence [GenBank: NG_007664] was used as reference for design of polymerase chain reaction (PCR) primers; Exons 4 and 5 were amplified as one PCR fragment.
